# Tagatose attenuates streptococcus mutans-induced dental caries by modulating oral microbiota and inflammatory responses in mice

**DOI:** 10.1186/s12866-026-04734-0

**Published:** 2026-01-22

**Authors:** Lili Zhao, Huakai Wang, Chengyi Miao, Qianqian Chen, Weiwei Wang, Wenjing Ma, Wenhui Zhang, Qingyu Wen, Ran Wang, Wanli Zhang, Wei Xiong

**Affiliations:** 1 Food Laboratory of Zhongyuan, Luohe, 462300 China; 2 College of Food Science and Technology, Henan University of Technology, Zhengzhou, 450001 China; 3 Key Laboratory of Precision Nutrition and Food Quality, Department of Nutrition and Health, China Agricultural University, Beijing, 100083 China

**Keywords:** Tagatose, Dental caries, Oral microbiota, Inflammatory factors, Streptococcus mutans

## Abstract

**Background:**

Dental caries is one of the most common chronic infectious oral diseases worldwide, leading to destruction of dental hard tissues and is associated with systemic diseases. Traditional caries prevention methods have limitations in terms of their applicability to specific populations or potential side effects. Therefore, exploring new, green, safe, and efficient caries prevention substances holds significant public health implications.

**Methods:**

This study employed a mouse experimental caries model induced by *Streptococcus mutans* (*S. mutans*), combined with 16 S rRNA sequencing analysis of oral microbiota dynamics and detection of inflammatory factors to evaluate the anti-caries effects of tagatose.

**Results:**

The results showed that tagatose intervention significantly reduced the severity of dental caries in mice in a dose-dependent manner. It inhibited cariogenic bacteria (*Staphylococcus*), promoted beneficial bacteria (*Streptococcus*), and regulated inflammatory factors (inhibiting pro-inflammatory factors such as IL-17, IL-6, and IFN-γ, and promoting anti-inflammatory factors such as IL-10 and IL-4), with lower concentrations showing better efficacy than xylitol.

**Conclusions:**

In summary, tagatose exhibits remarkable potential in the field of caries prevention, providing new scientific evidence and insights for the further development of natural anti-caries products based on tagatose.

## Background

Dental caries is a prevalent public health issue with significant implications for systemic health and well-being, largely due to its high global prevalence and potential to cause tooth loss [1]. This disease is initiated by cariogenic bacteria within dental plaque, which metabolize dietary carbohydrates to produce acids that lead to demineralization of tooth structure [2, 3]. The development of caries begins with early-stage enamel alterations, clinically observed as shallow pits or white spot lesions [4]. Without effective intervention, continued acid production leads to progressive demineralization, cavity formation, and ultimately, the destruction of dental hard tissues [5, 6]. Once caries develops, the standard treatment involves removing the diseased tissue and restoring the tooth with artificial filling materials [7]. Although this technique approach can repair structural defects, restorations are generally less aesthetic and functional than natural tooth structure, and may introduce long-term health and economic concerns [8]. Therefore, implementing effective strategies to control early caries is essential to preserving oral health and preventing irreversible damage.

Currently, most commercial products used for caries prevention rely on remineralizing agents or antimicrobial agents. Numerous studies have confirmed the efficacy of fluoride and calcium in preventing caries [9, 10]. However, concerns have been raised regarding potential fluoride toxicity (fluorosis) and allergic reactions. Similarly, locally applied antibacterial agents like chlorhexidine gluconate are widely used [11, 12], yet they are also associated with adverse effects, including allergic responses and irritation [13, 14]. In contrast, tagatose, a rare sugar with a sweet taste and low caloric value, offers a promising alternative. Replacing sucrose with tagatose may help reduce the risk of dental caries as well as metabolic disorders such as diabetes, which are linked to excessive consumption of high-calorie sugars [15–18]. Beyond its established role as a non-cariogenic sweetener, tagatose has been shown to influence the gut microbiota metabolome-particularly in the cecum and colon of pigs, increasing the production of beneficial short-chain fatty acids such as butyrate and valerate [19]. More relevant to oral health, tagatose can inhibit the growth of *S. mutans* [20] and interfere with bacterial metabolism and oral biofilm formation, thereby contributing to the prevention of periodontal disease and dental caries [17, 21]. These findings suggest that tagatose holds promise as a viable alternative to sucrose for daily caries prevention. Nevertheless, studies regarding its impact on other oral bacteria and its specific anti-caries efficacy remain limited.

To comprehensively evaluate the caries-preventing potential of tagatose and its systemic impact on the oral microbiome, this study employed a classic rodent dental caries model. Although in vitro biofilm models have value in the initial screening of antibacterial agents, they cannot simulate key physiological factors such as host dietary intake, salivary secretion, immune response, and the complex and variable microenvironment within the oral cavity. The selection of this in vivo model was based on the following considerations: Firstly, it allows us to assess the long-term effects of oral intake of tagatose on established, multi-species cariogenic biofilms in a complete biological system; Secondly, it can reveal the association between the oral microbiome structure and the local gingival tissue inflammatory state under tagatose intervention, which is difficult to achieve with in vitro models; Finally, this model can provide morphological evidence of caries formation and directly link microbial group changes with the actual disease phenotype. Therefore, this study aims to provide mechanistic insights beyond the simple antibacterial effect regarding how tagatose inhibits caries through regulating host-microbe interactions by utilizing the in vivo model of mouse dental caries.

## Methods

### Materials

Tagatose (99.98%) was purchased from Henan Yiheng Yuan Biotechnology Co., Ltd. (China). Agar powder, phosphate-buffered saline (PBS), and brain heart infusion (BHI) were purchased from Beijing Solabo Technology Co., Ltd. (Beijing, China). *Streptococcus mutans* (*S. mutans*, ATCC 25175) was purchased from the Shanghai Microbial Culture Collection Center (Shanghai, China).

### Preparation of bacterial samples

*S. mutans* were cultured in Brain heart infusion (BHI) medium at 37 °C. Prior to the experiment, all strains were stored at −80 °C. After activation, *S. mutans* was cultured at 37 °C for 12 h, then centrifuged at 10,000 g for 10 min to collect the cells [22]. After washing three times with PBS, the bacterial concentration was adjusted to 5 × 10⁸ CFU/mL by measuring the absorbance at 600 nm (OD_600_), and confirmed by counting on BHI solid culture medium via serial dilution. The suspension was prepared fresh for each use.

### Experimental design

Animal experiments were conducted in accordance with the China Agricultural University Guidelines for the Care and Use of Laboratory Animals and were approved by the China Agricultural University Institutional Animal Care Committee (No. AW42405202-1–08). Mice were purchased from Beijing SPF Biotechnology Co., Ltd (Beijing, China). After a one-week adaptation period, a classic mouse dental caries model was established following the methods described by Culp DJ et al. [23]. The specific procedure was as follows: 60 male SPF BALB/c mice aged 6 weeks with an average weight of 22.3 ± 1.8 g were randomly divided into 6 groups, with 10 mice in each group, including the control group (Control), the model group (Model), the xylitol group (Xylitol), the low-dose tagatose group (L-Tagatose), the medium-dose tagatose group (M-Tagatose), and the high-dose tagatose group (H-Tagatose). For the first 5 days, mice other than the Control group were inoculated daily with 5.0 × 10⁸ CFU of live *S. mutans* for caries modeling. Subsequently, mice in the Control group were fed a standard diet and sterile water; mice in the Model group were fed a caries-inducing diet and 10% (w/v) sterile sucrose water; mice in the Xylitol group were fed caries-inducing diet and 5% sterile xylitol sugar water (5% SX); mice in the L-Tagatose, M-Tagatose, and H-Tagatose groups were fed caries-inducing diet and 2%, 5%, and 8% sterile tagatose sugar water. The experiment lasted for 5 weeks, during which water and feed intake were strictly controlled to ensure that each group received the same volume and weight of water and feed. After the experiment was completed, the animals were fasted overnight and then placed in an induction room. They were subjected to inhalation anesthesia (a mixture of 2% isoflurane and 98% oxygen) until the foot reflexes were completely lost and breathing stopped. To ensure death, cervical dislocation should be performed immediately after breathing stops, followed by collection of blood, organ tissue, and tooth samples for further analysis.

### Dental sampling and scoring

After execution, the heads of mice were collected and boiled for 15 min. All oral cavities were then surgically dissected, and jawbones were obtained. This jawbone was stained with 0.4% murexide (Sigma-Aldrich) for 12 h, rinsed, and half-sliced along the occlusal surfaces of the maxillary and mandibular molars using a diamond cutter. Subsequently, the molars were visualized and estimated by standard methods under a stereomicroscope [24].

### HE staining

The tissues were fixed with 4% paraformaldehyde. All fixed specimens were clipped, dehydrated, embedded in paraffin, and sliced. The sections were stained with hematoxylin and eosin (HE) according to a published method [25].

### Serum inflammatory factors

ELISA was conducted to determine mouse IL-6, IFN-γ, IL-17, TNF-α, IL-10, and IL-4 (Nanjing Jiancheng Bioengineering Institute, Nanjing, China), according to the manufacturers’ instructions.

### 16S rRNA gene sequencing and microbial bioinformatics analysis

To assess microbial community composition, supragingival dental plaque samples were collected from the exposed tooth surfaces available to the mice during the last 3 days of the experiment using a sterile Gracey spatula. The 3-day microbial samples from each mouse were pooled according to a method to assess bacterial diversity and community abundance [26]. Mixed plaque samples were stored at −80 °C until genomic DNA extraction. Microbial DNA was isolated from the oral swab samples using the E.Z.N.A@soil DNA Kit (Omega Bio-Tek, Norcross GA, USA) according to the manufacturer’s instructions [27]. DNA concentration and purity were determined using 1% agarose gel electrophoresis and the NanoDrop™ 2000 spectrophotometer (Thermo Fisher Scientific, Waltham, MA, USA). Bacterial 16 S rDNA (V3–V4) was amplified using primers 338 F (5′-ACTCCTACGGGAGGCAGCAG-3′) and 806R (5′-GGACT ACHVGGGTWTCTAAT-3′) on an ABI GeneAmp^®^ 9700 PCR thermal cycler (Applied Biosystems, Foster City, CA, USA). PCR products were extracted from 2% agarose gels and purified using the AxyPrep DNA Gel Extraction Kit (Axygen Biosciences, Union City, California, USA) according to the manufacturer’s instructions, followed by quantification using the Quantus™ Fluorometer (Promega Corporation, Madison, Wisconsin, USA). Pair-end sequencing of purified amplicons mixed at equimolar concentrations was performed using the Illumina MiSeq PE300 platform (Illumina, San Diego, California, USA) following the standard protocol established by Majorbio Bio-Pharm Technology Co. Ltd. (Shanghai, China). Raw 16 S rRNA gene sequencing reads were demultiplexed, quality-filtered (using fastp version 0.20.0), and merged (using FLASH version 1.2.7). Operational taxonomic units (OTUs) were clustered using UPARSE version 7.1 with a similarity threshold of 97%; chimeric sequences were identified and removed. Microbial community α diversity was estimated using Chao and Shannon indices with QIIME 2 (version 2020.6). Principal coordinate analysis (PCoA) was performed using the arithmetic mean Bray–Curtis distance to visualize the complexity and diversity of bacterial community structure. Bacterial abundance was calculated as percentage abundance at taxonomic levels from phylum to genus. Kruskal–Wallis H test bar charts were used to identify differences in dominant bacterial communities between groups.

### Statistical analysis

The normality of the data distribution was verified with the UNIVARIATE procedure in SAS 9.2. For the assessment of statistical significance, a one-way ANOVA was employed, followed by Tukey’s test for multiple comparisons where appropriate. Differences were considered statistically significant at *p* < 0.05. Results are expressed as mean ± SEM. Spearman’s correlation analysis between bacterial profiles and serum indices was performed, and a heat map was generated using Origin 2021 (OriginLab, Northampton, MA, USA).

## Results

### Organ section analysis

**Fig. 1:**
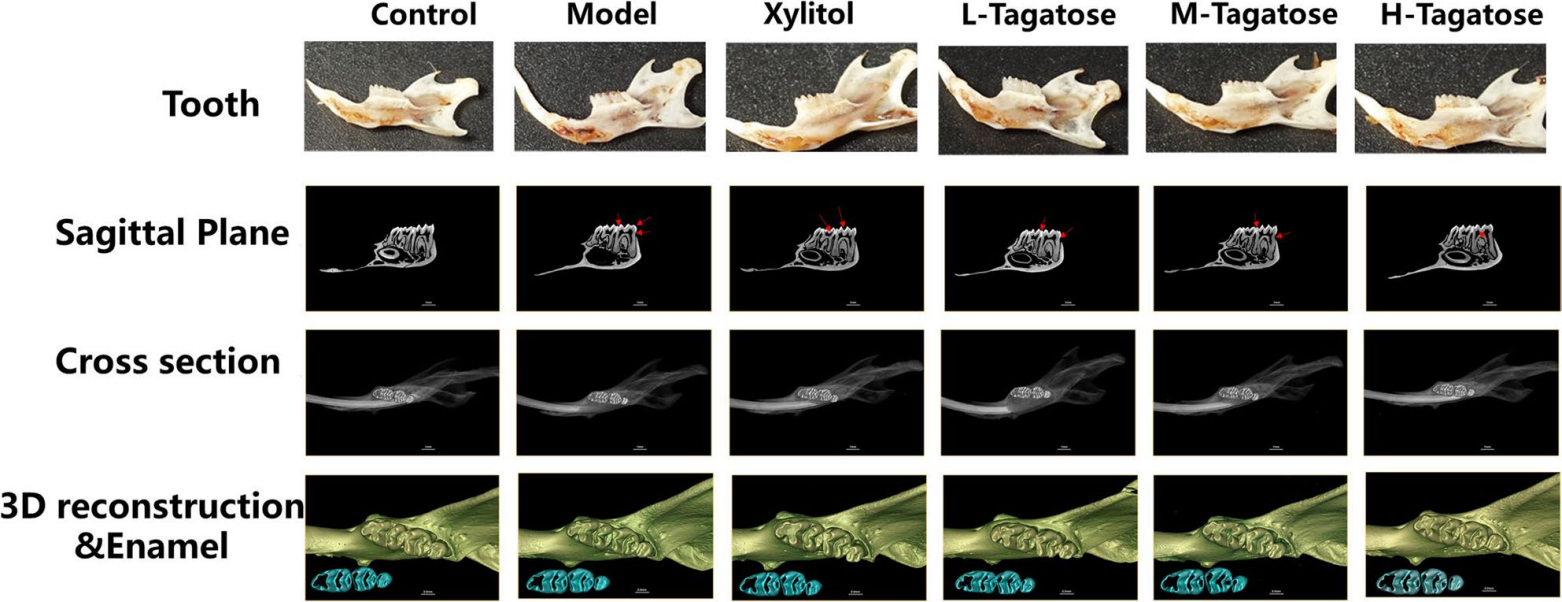
Effects of different treatments on dental caries, evaluated through micro-CT analysis. From top to bottom are images of teeth from different treatment groups; cross-sections (red arrows indicate caries lesions) and sagittal sections of molars analyzed via micro-CT; 3D reconstruction of maxillary molar micro-CT images; enamel (blue) in maxillary molar micro-CT images was distinguished by setting the density threshold above 4500 Hounsfield units

As shown in Fig. 1, The Control group exhibited uniformly white teeth with no noticeable discoloration or structural damage, indicating that the baseline diet posed no caries risk. The Model group showed widespread light brown spots on the enamel surface, with noticeable depressions in some areas, suggesting that a high-sugar environment significantly accelerates caries progression. The Xylitol group exhibited approximately 50% reduced caries severity compared to the model group, manifested as localized yellow spots. The tagatose intervention group showed a dose-dependent protective effect: the H-Tagatose group had a caries rate of 20%, the M-Tagatose group showed only scattered white demineralization, and the L-Tagatose group maintained high tooth integrity with only minor discoloration at the edges.

To further quantify the preventive effect of tagatose on dental caries in mice, micro-CT analysis was conducted to assess the depth and severity of caries lesions in different groups. Acid-producing bacteria-induced tooth demineralization is one of the most important characteristics of dental caries and can be observed via micro-CT for analyzing caries lesions (Fig. 1). The sagittal section displays the longitudinal anatomical structure of the sample, reflecting tissue morphological integrity; transverse sections assess the extent of internal damage (e.g., cavity size, enamel defects); green tones in 3D reconstructions indicate the extent of three-dimensional structural damage, while blue-green tones in enamel defects reflect enamel surface texture and mineral deposition status. Experimental results indicate that a high-sugar diet accelerates the progression of dental caries, with enlargement of carious lesions in sagittal sections and loss of repair signals in 3D reconstructions, and its destructive effect on enamel is irreversible. Based on corresponding sagittal section images, the tagatose group had fewer enamel demineralization sites (shaded areas marked with red arrows). The efficacy of tagatose intervention increases with concentration, with the H-Tagatose group approaching the control group level in both sagittal and transverse sections. However, in other groups, the number and area of demineralized sites in pits and fissures were more widespread, even reaching the pulp chamber, indicating severe tooth tissue damage in these groups. The xylitol group exhibited weaker caries severity than the model group, but its preventive efficacy was significantly weaker than that of tagatose at the same concentration.

### Biosafety assessment

**Fig. 2:**
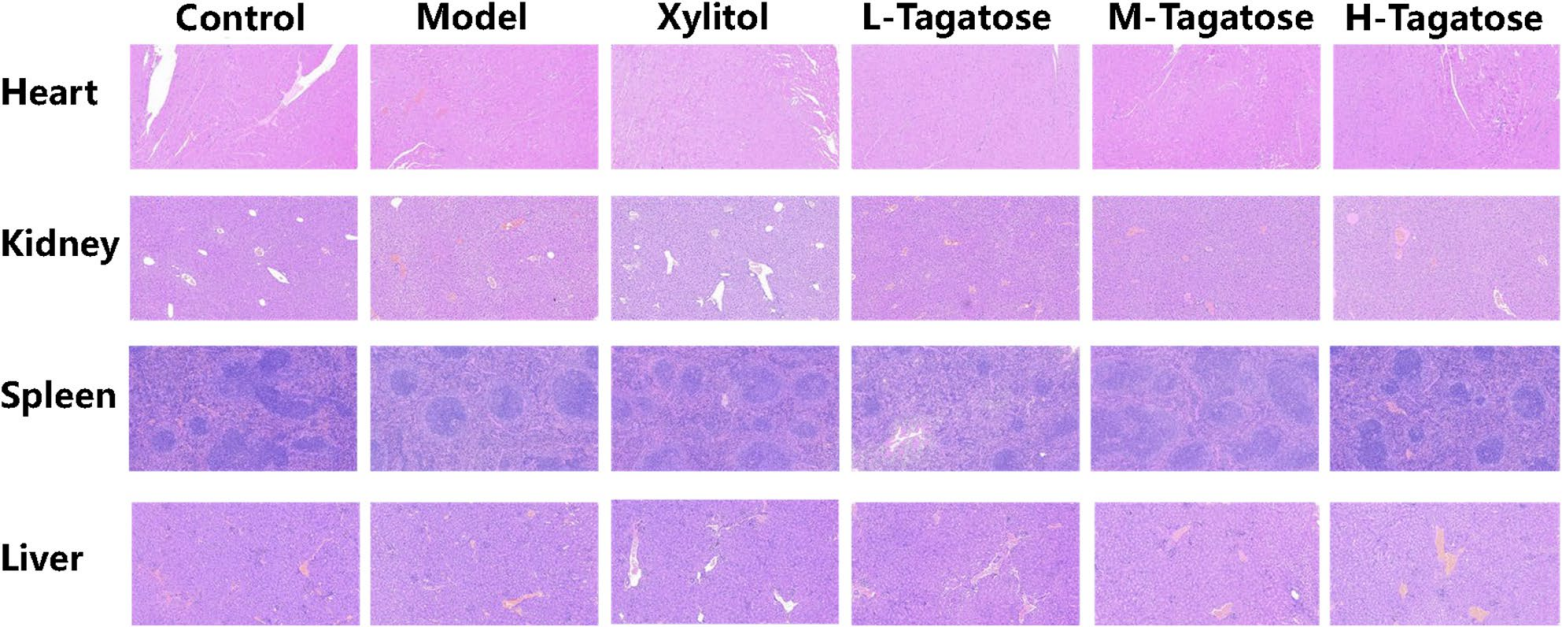
The HE sections of mouse heart, kidney, spleen, and liver tissues

Biological safety is a prerequisite for food ingredients. Using histopathological sectioning techniques, morphological changes in the heart, kidney, spleen, and liver organs of mice in the Control group, Model group, Xylitol group, L-Tagatose group, M-Tagatose group, and H-Tagatose group were compared and analyzed. Sections were stained with hematoxylin and eosin (HE), with purple staining indicating the distribution of cell nuclei and cytoplasm, and the depth of color and texture details characterizing tissue structural integrity. As shown in Fig. 2, HE staining of mouse organ tissues is a critical area in the treatment of gingivitis, but no significant tissue damage was observed in any of the treatment groups. This indicated that sucrose, xylitol, and tagatose do not damage mouse body tissues during treatment, demonstrating good biosafety.

### Changes in inflammation levels in mice

**Fig. 3:**
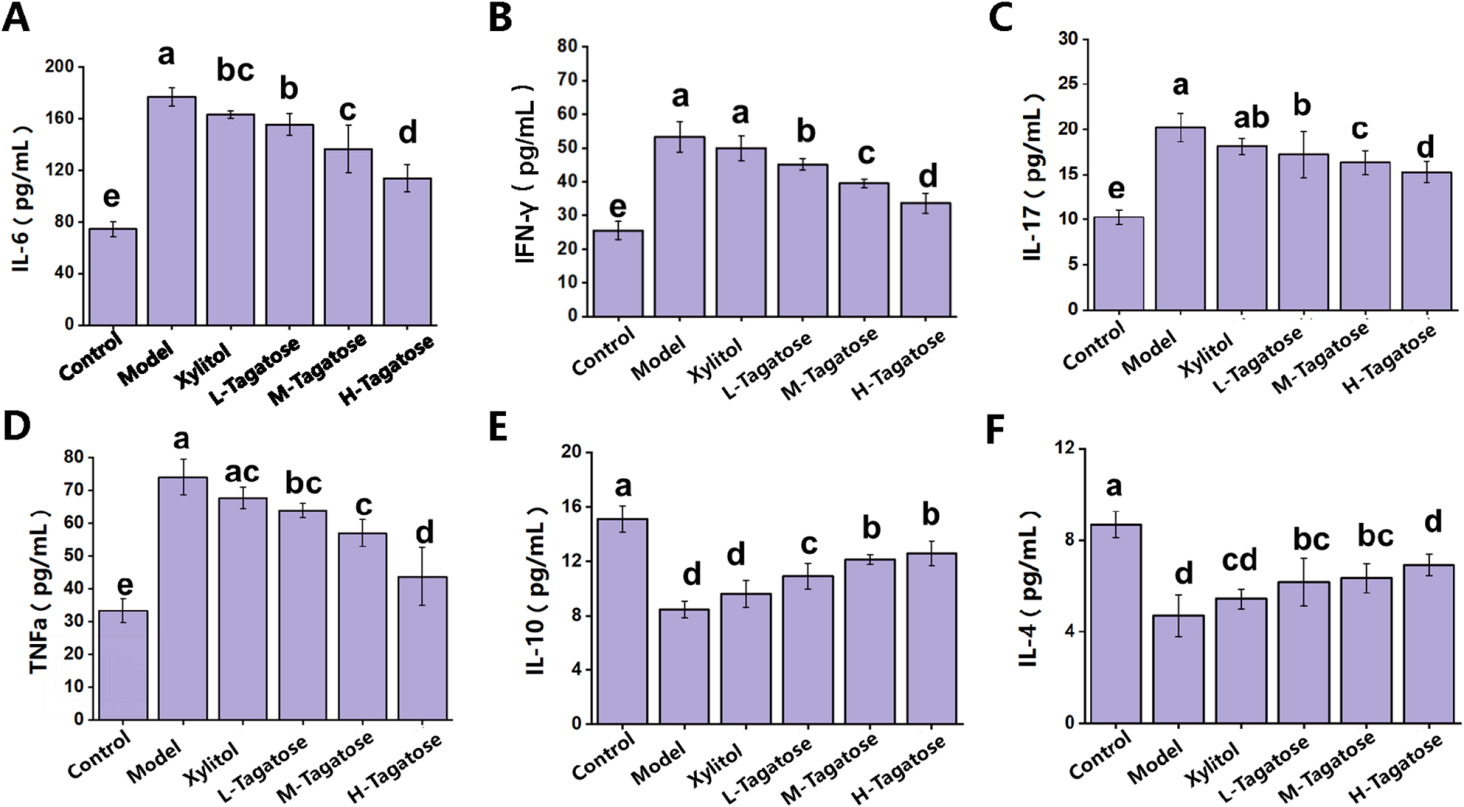
Effects of tagatose on inflammatory factors in mice blood. **A** IL-6; **B** IFN-γ; **C** IL-17; **D** TNF-α; **E** IL-10; **F** IL-4. Bars with different letters are significantly different (*P* < 0.05) as determined by Tukey’s HSD post-hoc test following one-way ANOVA. Values are expressed as mean ± SEM, *n* = 5 per group

As shown in Fig. 3, pro-inflammatory cytokine levels were markedly elevated in the model group, indicating a pronounced inflammatory response associated with *S. mutans*-induced caries. Specifically, IL-6 increased to 176.87 pg/mL in the model group compared with 74.38 pg/mL in the control (*p < 0.05*). Tagatose treatment reduced IL-6 in a dose-dependent manner, with low-, medium-, and high-dose groups showing 155.44, 136.55, and 113.80 pg/mL, respectively; the high-dose group approached the control level and was markedly lower than the xylitol group (163.19 pg/mL). IFN-γ exhibited a similar pattern, decreasing from 53.23 pg/mL in the model group to 39.46 and 33.61 pg/mL in the medium- and high-dose tagatose groups, respectively. IL-17 also declined from 20.31 pg/mL in the model to 15.26 pg/mL in the high-dose group, which was close to the control (10.25 pg/mL) and significantly lower than xylitol (18.13 pg/mL). TNF-α showed a clear dose-dependent decrease from 74.02 pg/mL in the model group to 43.71 pg/mL in the high-dose group.

For anti-inflammatory cytokines, IL-10 and IL-4 were markedly suppressed in the model group (8.21 and 4.35 pg/mL). Tagatose supplementation significantly restored their levels: IL-10 increased to 12.13 and 12.58 pg/mL in the medium- and high-dose groups, approaching the control (15.11 pg/mL) and exceeding the xylitol group (9.61 pg/mL). Similarly, IL-4 rose progressively with dose, reaching 6.91 pg/mL in the high-dose group compared with 5.43 pg/mL in the xylitol group.

### Composition of the oral microbiota of mice

**Fig. 4:**
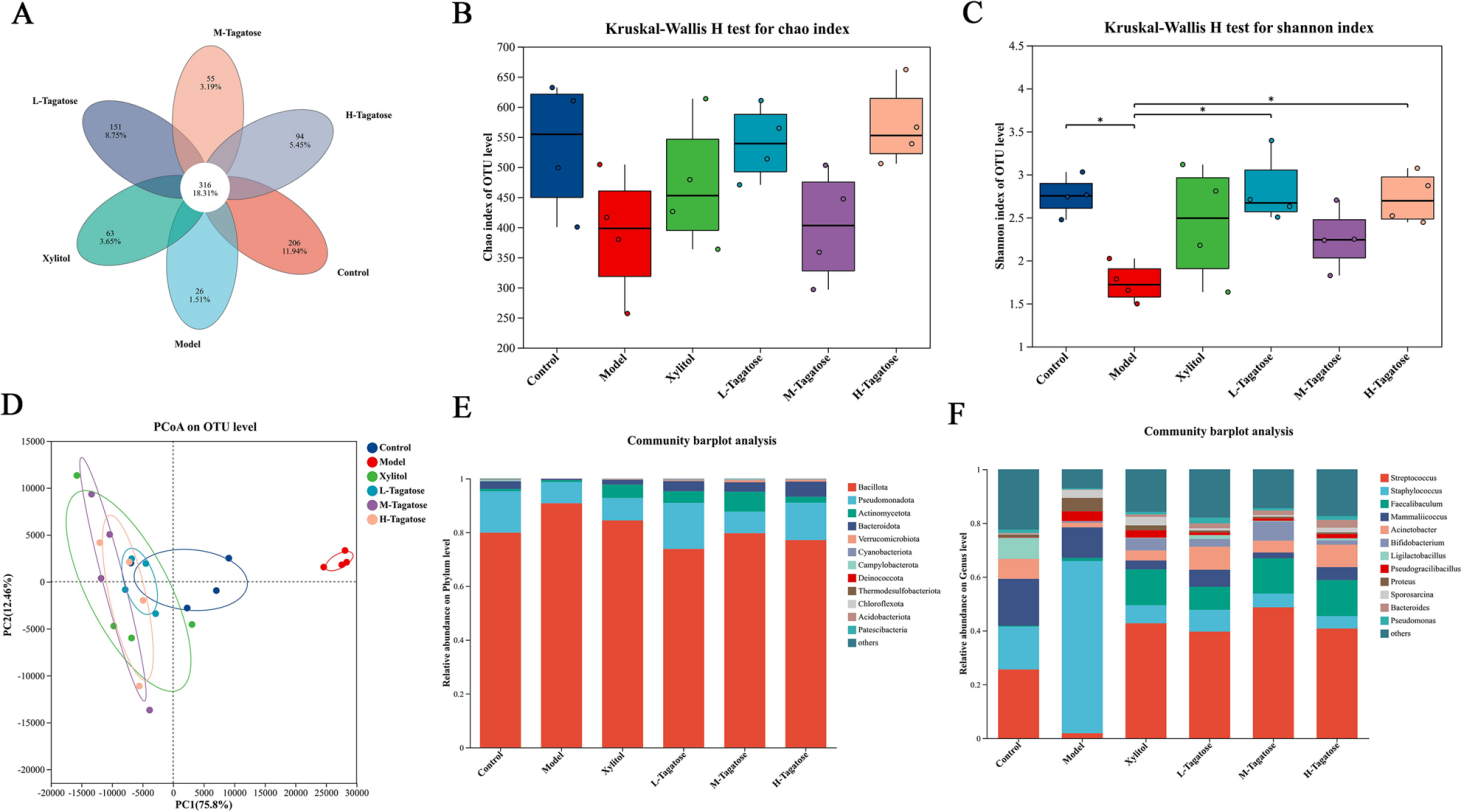
The effect of tagatose on the composition of the oral microbiota in mice. **A** Venn diagram; **B** Chao index; **C** Shannon index; **D** principal component analysis; **E**, **F** bacterial phylum and genus levels. Values are expressed as mean ± SEM, *n* = 5 per group

Figure 4 shows the composition and distribution of oral microbiota in each group. A total of 1,725 OTUs were identified in the six groups of oral microbial samples. Among these, 316 OTUs were present in all groups, indicating the existence of a core microbial community composition (Fig. 4A). There was no difference in the Chao index among different groups (Fig. 4B). Compared with the Model group, the Control, the L-tagatose, and the H-tagatose groups showed a higher Shannon index (Fig. 4C). The PCoA results showed clear separations betweent the groups, indicating that their gut microbial community structures were significantly different (Fig. 4D).

The relative abundance of the oral cavities of mice at the phylum and genus levels was shown in Fig. 4E and F. At the phylum level, Bacillota and Pseudomonadota were the dominant bacterial communities, followed by Actinomycetota, Bacteroidota, Verrucomicrobiota. At the genus level, compared with the control group, the abundance of *Streptococcus* was decreased while the abundance of *Staphylococcus* was increased in the model group. The xylitol group and tagatose groups reversed the abundance of *Streptococcus* and *Staphylococcus* compared with the Model group.

### Correlation analysis between inflammatory factors in mouse blood and bacterial spectrum

**Fig. 5:**
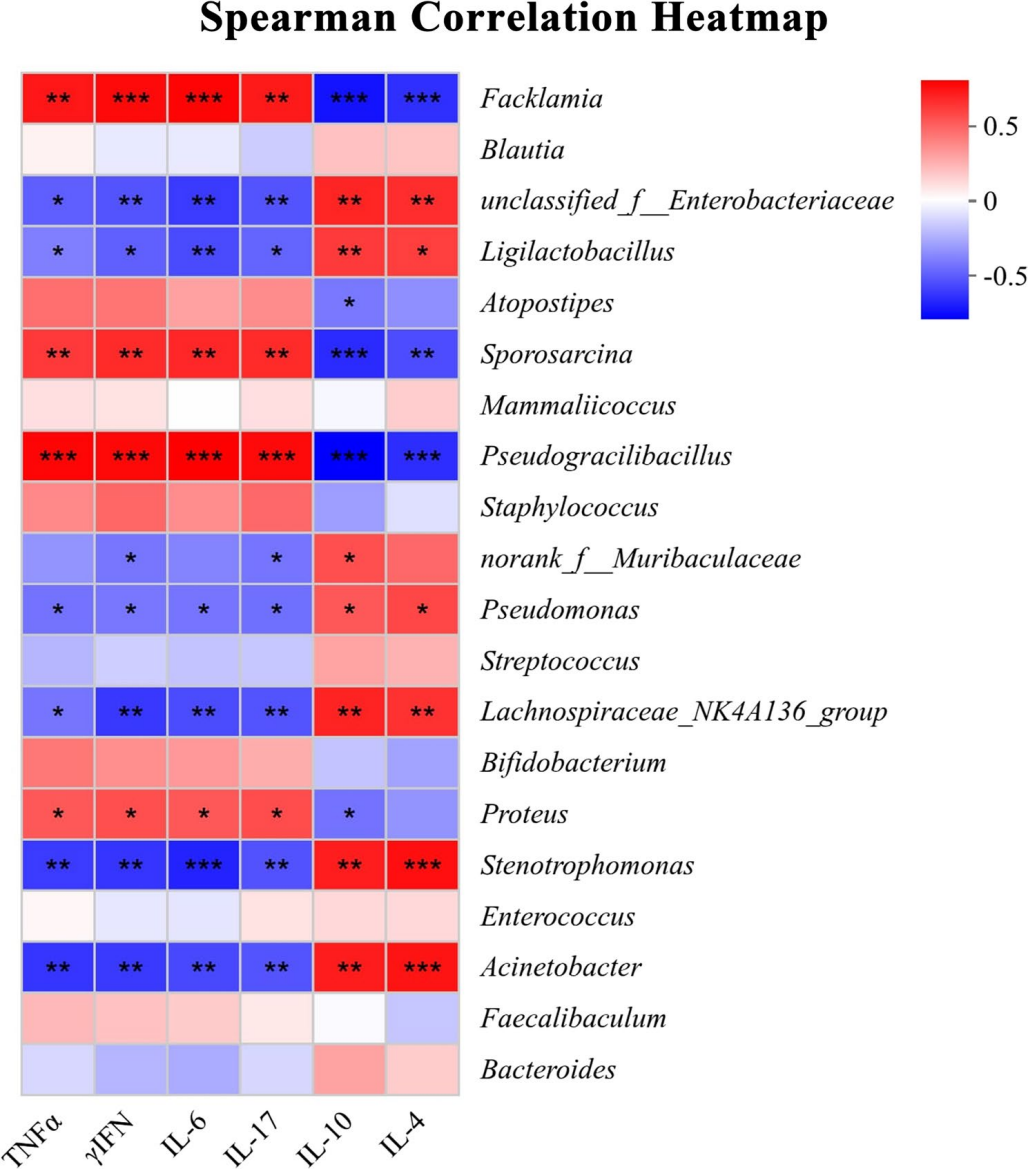
The correlation heat map depicts the association between bacterial profiles and serum indices. Correlations are color-coded, with red indicating positive correlations and blue indicating negative correlations. Significance levels are * *P* ≤ 0.05, ** *P* ≤ 0.01, and *** *P* ≤ 0.001

The correlation pattern between the 20 most abundant bacterial taxonomic groups and various serum markers is shown in Fig. 5. The abundance of *Facklamia*, *Sporosarcina*, *Pseudogulbuccali*, and *Proteus* was positively correlated with TNF-α, IFN-γ, IL-6, and IL-17, and negatively correlated with IL-10. Meanwhile, the abundance of *Facklamia*, *Sporosarcina*, and *Pseudogulbuccali* was negatively correlated with IL-4. Moreover, the abundance of *unclassified_f_Enterobacteriaceae*, *Ligiactobacillus*, *Pseudomonas*, *Lachnospiraceae_NK4A136_group*, *Stenotrophomonas*, and *Acinetobacter* was positively correlated with IL-10 and IL-4, while negatively correlated with TNF-α, IFN-γ, IL-6, and IL-17. Additionally, the abundance of *norank_f_Muribaculaceae* was negatively correlated with IFN-γ and IL-17, while positively correlated with IL-10.

## Discussion

Dental caries is a progressive lesion of the tooth’s hard tissue caused by the combined effects of various factors in the oral cavity, characterized by the demineralization of inorganic substances and the decomposition of organic substances, and evolving from color changes to the formation of substantive lesions [28]. Given the limitations of current caries treatments, novel approaches for safe and effective prevention and management are required. As a natural low-calorie sweetener, tagatose has demonstrated significant potential in preventing dental caries due to its non-cariogenic properties. In the present research, an in vivo trial was conducted to explore the caries-preventing effect of tagatose and its influence on oral microorganisms using a murine caries model induced by *S. mutans.*

Despite being the most-used reference standard for validating caries lesion depth, histological inspection (typically by stereomicroscopy) requires destructive sample preparation, which introduces variation. In line with the need for alternatives, Oliveira et al. demonstrated that micro-CT provides moderate confidence for in vitro caries detection on proximal tooth surfaces when compared to histology [29]. This study conducted micro-CT scans to explore the effect of tagatose on mouse caries. Our results showed that widespread light brown spots on the enamel surface, with noticeable depressions in some areas in the Model group, while the tagatose intervention group showed a dose-dependent protective effect. This finding was consistent with previous research, which includes a systematic review of randomized clinical trials that demonstrated the potential of tagatose to reduce the risk of dental caries [30]. This evidence shows that tagatose may be able to prevent dental caries caused by *S. mutans*.

Based on the histopathological analysis of key organs (heart, kidney, spleen, liver), this study provides crucial evidence for the biosafety of tagatose consumption in a murine model. The absence of significant morphological damage or structural alterations across all treatment groups—including the high-dose tagatose (H-Tagatose) group—demonstrates that its administration, at the tested levels, does not induce systemic toxicity. This finding is fundamental, as it establishes that the observed anti-caries effects in our study are not conflated with or offset by adverse physiological effects. The excellent tissue compatibility of tagatose, which was comparable to both the Control and Xylitol groups, strongly supports its profile as a safe food ingredient [31]. This biosafety, coupled with its previously discussed efficacy in mitigating *S. mutans*-induced caries, positions tagatose as a promising dual-action sugar substitute [32]. It offers the desired sweetening property while simultaneously exerting a protective effect against a primary caries pathogen without harming vital organs. Furthermore, the lack of tissue damage in the tagatose groups suggests that its modulation of the oral microbiome and its inhibitory action on *S. mutans* are likely localized to the oral cavity and do not stem from a systemic, and potentially harmful, mechanism [16]. In conclusion, the histopathological results confirm that the anti-caries benefits of tagatose are achieved with a high margin of systemic safety in this model. This strengthens the case for its potential application in functional foods and oral health products.

The analysis of key pro-inflammatory and anti-inflammatory cytokines provides crucial insights into the immunomodulatory mechanism by which tagatose exerts its protective effects in the *S. mutans*-induced caries model. The significant elevation of pro-inflammatory cytokines (IL-6, IFN-γ, IL-17, and TNF-α) coupled with a reduction in anti-inflammatory cytokines (IL-10 and IL-4) in the Model group indicates a potent Th1/Th17-driven inflammatory response against the cariogenic challenge. This imbalance is critical, as these cytokines can promote osteoclast activation and tissue destruction in the periodontium, exacerbating oral disease progression [33]. The dose-dependent restoration of this cytokine profile by tagatose supplementation—effectively suppressing pro-inflammatory mediators while elevating anti-inflammatory ones—demonstrates its capacity to rebalance the host’s immune response. This shift towards a more regulated, Th2/Treg-type response, characterized by increased IL-4 and IL-10, is likely a key factor in mitigating the immunopathologic damage caused by *S. mutans* [16]. Importantly, while xylitol showed a limited effect by reducing only IL-6, tagatose exhibited a broader and more potent immunomodulatory action. This suggests that the anti-caries efficacy of tagatose is not only due to its direct or microbiome-mediated antagonism against *S. mutans* but is also significantly contributed by its systemic modulation of the host’s inflammatory status, thereby controlling the destructive inflammatory sequelae of the infection.

Dysbiosis of the oral microbiota is a key driver of dental caries. The analysis of the oral microbial composition provides compelling evidence that tagatose supplementation can mitigate *S. mutans*-induced dysbiosis, thereby contributing to its observed anti-caries effects. An intriguing finding was the non-monotonic effect of tagatose on the Shannon diversity of the oral microbiota. While both low and high doses showed higher Shannon diversity compared to the Model group, the medium dose resulted in a significant decrease, which may reflect the complex competitive and cooperative dynamics within the microbial community. We speculate that the low-dose tagatose may slightly disturb the ecological niche of the pathogenic bacterial community dominated by the Streptococcus genus, creating limited space for the growth of some symbiotic bacteria. The medium-dose may most effectively inhibit the dominant pathogenic bacteria represented by *S. mutans*, but this strong inhibition may not simultaneously promote the colonization of other bacterial communities, instead temporarily reducing the uniformity of the community and failing to significantly increase the diversity index. the high-dose may trigger a more extensive ecological remodeling, not only inhibiting the pathogenic bacteria, but also its metabolic products or environmental changes (such as pH changes) may be more conducive to the growth of various bacteria that are tolerant or capable of utilizing new ecological niches, thereby restoring a higher community diversity. This phenomenon is consistent with the significant separation between groups shown in the PCoA analysis, emphasizing the importance of precise regulation of the intervention dose for guiding the microbial community to transition to the expected state [34]. At the phylum level, the enrichment of Bacillota and Pseudomonadota in the Model group is consistent with a state of pathogenic dysbiosis and inflammation driven by *S. mutans* infection. The ability of tagatose to restore the abundance of these phyla to levels comparable to the Control group indicates a reversal of this cariogenic state. One interesting finding worth exploring in depth is that, although *S. mutans* was inoculated during the modeling process, the overall relative abundance of *Streptococcus* in the model group showed a significant decrease. This may seem contradictory, but it actually reveals the complexity of microbial community intervention and the potential selectivity of tagatose action. First, it is necessary to clarify that the 16 S rRNA sequencing provides relative abundance data, reflecting the proportional relationship among the members of the community. Secondly, the *Streptococcus* genus is a functionally diverse group that includes pathogenic species such as *S. mutans*, as well as species like *Streptococcus salivarius* that are considered oral symbionts or beneficial bacteria [35]. We speculate that the intervention of xylitol and tagatose may have produced differential effects: it effectively inhibited the growth of cariogenic *Streptococcus*, including the *S. mutans*; at the same time, the changes in the oral environment (such as pH regulation) may have created ecological niches for certain non-cariogenic *Streptococcus* species. Moreover, the harmful bacteria (*Staphylococcus*) in the model group significantly increased, which may be the main reason for the overall decrease in the abundance of *Streptococcus*. These findings, combined with the lack of systemic toxicity observed in our histopathological analysis, strongly suggest that the anti-caries mechanism of tagatose involves a direct, localized modulation of the oral microbiome, effectively suppressing cariogenic pathogens and promoting a healthier microbial community structure without inducing adverse systemic effects [36]. Further metagenomic sequencing is necessary to analyze the specific changes in microbial species.

This study employed a classic mouse dental caries model to verify the overall potential of tagatose in preventing dental caries by regulating the oral microecology within a controllable in vivo system. We fully recognize that extrapolating the findings of this model to human clinical practice requires careful consideration of its boundaries. Firstly, from an ethical perspective, the choice of the in vivo model in this study stems from its irreplacability: the core objective is to explore the integrated effect of oral intake of tagatose in the “host-microbe-hard tissue” dynamic complete system, which involves system metabolism, immune interaction, and long-term ecological evolution, and is beyond the scope of existing in vitro models. We followed the 3R principles by using efficient classic models (optimization/Refinement) and multi-index analysis to minimize animal use (reduction/Reduction). Secondly, in terms of model characteristics, by inoculating cariogenic bacteria and inducing acute caries damage through a high-sugar diet, we provided an “extreme challenge” environment. This design can clearly reveal the upper limit of the action mechanism of tagatose; however, the observed potency is evidence of its biological potential under harsh conditions, rather than a direct efficacy prediction for human multifactorial, slow-progressing dental caries. Furthermore, regarding species differences, we acknowledge that the oral microbial composition of mice and humans is different, so the translational value does not lie in the direct correspondence of the microbiota, but in revealing the universal ecological principle of “suppressing pathogens by enriching endogenous non-cariogenic symbiotic bacteria”, which provides a core hypothesis for translational research [37–39]. Finally, in terms of exposure methods, continuous administration via drinking water (2–8%) differs from the local, short-term oral care product exposure (involving saliva clearance) in humans. Therefore, the dose used in this study should be regarded as a systematic proof-of-concept, and the optimal concentration and frequency for local application in humans need to be re-explorated in human biofilm models or clinical studies. In summary, within the ethical framework, this study successfully answered the fundamental question of whether tagatose can exert anti-caries effects in the living system through ecological regulation. Its core contribution lies in drawing a clear translational roadmap: first, validate and optimize its local application scheme in a human-derived biofilm model; second, based on the ecological regulation principle revealed in this study, explore in clinical research whether tagatose can enrich native bacteria with similar beneficial functions in human oral cavity and evaluate its clinical endpoints, thereby guiding the mechanism discovery towards application transformation.

## Conclusions

In the present study, we explored the effects of tagatose on dental caries and oral microbiota using a mouse model induced by *S. mutans*. The results demonstrated that tagatose intervention exerted a significant anti-caries effect in a dose-dependent manner. Additionally, tagatose regulated the balance of the oral microbiota (inhibiting overgrowth of harmful bacteria and promoting proliferation of beneficial bacteria) and modulated inflammatory factors (suppressing pro-inflammatory cytokines such as IL-6 and IFN-γ, while enhancing anti-inflammatory cytokines such as IL-10 and IL-4). These findings indicate that tagatose can not only function as a sweetener in food but also serve as a natural anti-caries ingredient in oral care products. Future studies should extend intervention periods to investigate the dynamic changes of key genera (e.g., *S. mutans*, *Streptococcus sanguinis*), develop anti-caries oral care products, and further explore the underlying mechanisms.

## Data Availability

The obtained metabarcoding sequences have been submitted to the Sequence Read Archive of National Center for Biotechnology Information under the accession number PRJNA1367214.

## Abbreviations

BHI: Brain Heart Infusion
Control: The Control Group
HE: Hematoxylin And Eosin
H-Tagatose: The High-Dose Tagatose Group
L-Tagatose: The Low-Dose Tagatose Group
Model: The Model Group
M-Tagatose: The Medium-Dose Tagatose Group
OTUs: Operational Taxonomic Units
PBS: Phosphate-Buffered Saline
PCoA: Principal Coordinate Analysis
S. mutans: Streptococcus mutans
Xylitol: The Xylitol Group
